# Improved Glucose-Stimulated Insulin Secretion by Selective Intraislet Inhibition of Angiotensin II Type 1 Receptor Expression in Isolated Islets of db/db Mice

**DOI:** 10.1155/2013/319586

**Published:** 2013-11-24

**Authors:** Zhen Zhang, Chunyan Liu, Zhenhua Gan, Xinyi Wang, Qiuyan Yi, Yanqing Liu, Yingzhijie Wang, Bin Lu, Hong Du, Jiaqing Shao, Jun Wang

**Affiliations:** ^1^Department of Endocrinology, Jinling Hospital, Southern Medical University, 305 Zhongshan East Road, Nanjing, Jiangsu Province 210002, China; ^2^Department of Cardiology, Jinling Hospital, Southern Medical University, 305 Zhongshan East Road, Nanjing, Jiangsu Province 210002, China

## Abstract

Recent evidence supported the presence of a local renin-angiotensin system (RAS) in the pancreas, which is implicated in many physiological and pathophysiological processes. We utilized small interfering RNA (siRNA) to investigate the effects of angiotensin II type 1 receptor (AT1R) knockdown on glucose-stimulated insulin secretion (GSIS) in isolated islets of db/db mice and to explore the potential mechanisms involved. We found that Ad-siAT1R treatment resulted in a significant decrease both in AT1R mRNA level and in AT1R protein expression level. With downexpression of AT1R, notable increased insulin secretion and decreased glucagon secretion levels were found by perifusion. Simultaneously, significant increased protein levels of IRS-1 (by 85%), IRS-2 (by 95%), PI3K(85) (by 112.5%), and p-Akt2 (by 164%) were found by western blot. And upregulation of both GLUT-2 (by 190%) and GCK (by 121%) was achieved after AT1R inhibition by Ad-siAT1R. Intraislet AT1R expression level is a crucial physiological regulator of insulin sensitivity of **β** cell itself and thus affects glucose-induced insulin and glucagon release. Therefore, the characteristics of AT1R inhibitors could make it a potential novel therapeutics for prevention and treatment of type 2 diabetes.

## 1. Introduction

Recent decades have seen a dramatic rise in the incidence and prevalence of type 2 diabetes mellitus (T2DM) throughout the world. The damage of pancreatic islet function plays a crucial role in the pathogenesis and progression of T2DM. However, current treatment of T2DM can not provide effective protection against islet failure. Interestingly, recent clinical researches had shown that RAS blockade by ACE inhibitor (ACEI) or angiotensin receptor blocker (ARB) could reduce the onset of diabetes in people at high risk by 14%–34% [1–4]. The mechanisms underlying this protective effect appear to be complex and may involve improvement of both insulin sensitivity and insulin secretion. However, the detailed mechanisms are still unknown.

 RAS components, such as angiotensinogen (AGT), angiotensin converting enzyme (ACE), angiotensin II (AngII) and type 1 and type 2 angiotensin II receptors (AT1R and AT2R), had been found in islets [5, 6]. Evidence suggests that the local pancreatic islet RAS performs multifactorial activities in structure and function of islet, including cell proliferation, apoptosis, oxidative stress, inflammatory responses, and glucose-stimulated insulin secretion, and these regulatory functions are probably mediated via AT1R [7]. Such a local islet RAS is subject to overactivation by diabetes and thus drives islet fibrosis and reduces islet blood flow, oxygen tension, and insulin biosynthesis. Kampf et al. demonstrated that endogenous levels of Ang II exerted detrimental effects on islet blood perfusion in transplanted mouse islets [8]. Moreover, overactivation of an islet RAS may accelerate the synthesis of reactive oxygen species, aggravate oxidative stress-induced *β*-cell dysfunction, and apoptosis and thus contribute to the islet failure seen in type 2 diabetes [9]. Our previous studies found that candesartan treatment could improve glucose tolerance with the preservation of *β*-cell mass and morphology in db/db mice [10, 11]. However, it is unclear whether or not these benefits are dependent on the changes of circulating RAS components. So selective inhibition of AT1R expression in islet could reveal the definite role of intraislet RAS in glucose homeostasis.

 The insulin receptors and their substrates (IRS-1 and IRS-2) have been proved to be expressed in the *β*-cell [12–14]. By the insulin signaling pathway, these molecules could impact GSIS of *β*-cell [15, 16]. Accumulated data have demonstrated that local RAS expressed in peripheral tissues is overactivated in the state of insulin resistance, and the effects of RAS blockade on insulin resistance have been proved [17, 18]. As there is evidence for insulin signaling molecules expression in islets itself and because RAS is implicated in the pathogenesis of insulin resistance, it is possible that local RAS expressed in islets may affect GSIS through insulin signaling pathway of *β*-cell.

In the present study, we aim to explore whether intrinsic RAS in islet is involved in glucose-stimulated insulin secretion by affecting insulin sensitivity of *β*-cell itself via RNA interference technique which can inhibit the expression of intraislet AT1R effectively and specifically.

## 2. Materials and Methods

### 2.1. Islet Isolation and Culture

Five eight-week old female db/db mice and five age and gender matched nondiabetic littermates db/m mice were obtained from the Animal Experiment Center of Jingling Hospital. Mice were anesthetized with pentobarbital (Nembutal, Abbot Laboratories). After clamping the common bile duct at a point close to the duodenal outlet, pancreas was injected through the pancreatic duct with 2 mL Krebs-Ringer bicarbonate buffer (KRBB: 129 mmol/L NaCl, 5 mmol/L NaHCO3, 4.8 mmol/L KCl, 1.2 mmol/L KH2PO4, 1.2 mmol/L MgSO4, 0.2% BSA, 10 mmol/L Hepes, 2.5 mmol/L CaCl2, and 2.8 mmol/L glucose at pH 7.4) containing 1.5 mg/mL of collagenase (Worthington, Biochemical Co., St. Louis, Missouri). The swollen pancreas was removed and incubated at 37°C for 40 min. The digested pancreas was shaken and washed with ice-cold HBSS four times, and islets were handpicked under a stereomicroscope cultured in RPMI 1640 medium at 37°C in a humidified atmosphere (5% CO2, 95% air). For both batch incubation and perifusion studies, the islets were pre-incubated for 30 min in KRBB (1.4 mmol/L glucose) at 37°C, 5% CO2, and saturated humidity.

### 2.2. Isolation of Total RNA and Real-Time Reverse Transcription PCR of AT1R

Total RNA was extracted from islets by the TRIzol reagent according to the manufacturer's protocol (Invitrogen). For quantitative real-time PCR, the first strand cDNA was synthesized from 300 ng of total RNA using the oligo (dT) primer and MMLV reverse transcriptase (Invitrogen). Samples were subjected to quantitative amplification using the TaqMan probe and primer sets for mice AT1R. PCR amplification was performed in a total volume of 10 *μ*L containing 30 ng of cDNA, 900 nM of each primer, 250 nM of the respective probe, and 6 *μ*L of Taq Man Universal PCR Master Mix. Real-time PCRs (95°C for 15 s, 55°C for 20 s, and 72°C for 20 s × 35 cycles) were performed in an ABI-Prism 7700 sequence detector system (Applied Biosystems, Foster City, CA). Different cDNA samples were normalized using primer sets to the housekeeping gene *β*-actin. Primers were as follows: AT1R, 5′-AGCTACAACAAGGCAAGG-3′ and 3′-TAGAAGGCACAGTCGAGG-5′; *β*-actin, 5′-TGTTGTCCCTGTATGCCTCTGGTC-3′ and 3′-ATGTCACGCACGATTTCCCTCTCA-5′. The fold changes were calculated by using the comparative threshold cycle method.

### 2.3. Cell Culture

INS-1 cells were grown in monolayer cultures in RPMI 1640 complete medium at 11.1 mmol/L glucose supplemented with 10% (w/v) fetal bovine serum, 10 mmol/L HEPES, 2 mmol/L L-glutamine, 1 mmol/L sodium pyruvate, and 50 *μ*mol/L *β*-mercaptoethanol at 37°C in a humidified atmosphere (5% CO2, 95% air). For transfection experiments, the cells were seeded in 75-cm^2^ flasks at 4 × 10^6^ cells 2 days prior to transfection and were at 60–70% confluency at the time of the transfection.

### 2.4. Short Hairpin RNA-Mediated Gene Suppression

shRNAs directed against mice AR1R (GenBank accession number NM_030985) were designed according to Ambion (Austin, TX) siRNA design guidelines. Briefly, from 5′- to 3′-end, the whole length of shRNA was sequentially composed of the BamHI restriction site, the 19-nucleotide antisense gene-targeting sequence, TTT TTT ending transcription sequence, and HindIII restriction site. Three different shRNAs candidates (siAT1R1, siAT1R2, and siAT1R3) were designed and tested for their potency to decrease the targeted gene expression. A duplex with no known target (GAG ACC CTA TCC GTG ATT A) was used as control (siControl). All of the oligonucleotides were synthesized and purified. After annealing, the double-stranded oligonucleotides were ligated by self-formed restriction sites for BamHI and HindIII in pSilencer 2.0 vector (Ambion). The siRNAs were transfected into INS-1 cells using Lipofectamine PLUS at a concentration of 5 *μ*g of DNA for 6 × 10^6^ cells. Three days after transfection, INS-1 cells were harvested for RNA and protein. AT1R expression was confirmed by qRT-PCR and immunoblot analysis as described above. The most efficient one (siAT1R2) was chosen for subsequent experiments. Relative to the start codon, the 5′ ends of the target correspond to mice AT1R nucleotide 540 (GCGTCTTTCTTCTCAATCT). 

### 2.5. Recombinant Adenovirus Construction

Recombinant adenoviruses containing the siATR12 (Ad-siATR1) or the siControl (Ad-siControl) sequences described above were constructed using AdEasy System. Briefly, for each pSilencer-based clone, the siRNA expression cassette was excised and ligated into linearized adenoviral shuttle vector pAdTrack. Subsequently, 1 *μ*g recombinant PmeI-linearized pAd-siATR1 was transfected into *Escherichia coli* BJ5183 cells with an adenoviral backbone plasmid, pAdEasy-1. Recombinants were selected and successful recombination was determined by restriction endonuclease analysis. The linearized recombinant adenoviral construct was transfected into 293 cells and high-titer viral stocks were prepared. Viral titers were determined by plaque assay and expressed as plaque-forming units per mL (pfu/mL). The viral titers of Ad-siATR1 and Ad-siControl were 3.6 × 10^9^ pfu/mL and 2.9 × 10^9^ pfu/mL, respectively. 

### 2.6. Gene Silencing in Islets of Langerhans

Islets of db/db mice and db/m mice in aliquots of 50 islets per well of a six-well plate in 2 mL medium were divided into three groups: (1) Ad-siAT1R group, islets were treated with Ad-siAT1R for 20 h at 2,000 plaque-forming units/islet; (2) Ad-siControl group, islets were treated with Ad-siControl; (3) Control group, Mock transduced islets. Mock infected islets were not exposed to virus during the incubation period and were not incubated with any vectors. After removal of virus-containing medium, islets were cultured for an additional 72 h with medium changes every 24 h. GSIS was measured. Subsequently, islets were collected and lysed for analysis of AT1R expression as described above.

### 2.7. Islet Perifusion

Kinetics of insulin release in vitro was studied by the perifusion system. Size-matched 50 islets were placed in each column. Then the columns were gently closed with the top adaptors, immersed in vertical position and controlled temperature in the water bath at 37°C. The perifusion medium was maintained at 37°C in a water bath. And all columns were perfused in parallel at a flow rate of 0.5 mL/min with KRB (2.8 mmol/L glucose) at 37°C. After 60 min static incubation with KRB (2.8 mmol/L glucose), the islets were stimulated in the continuous presence of a high concentration of 16.7 mmol/L glucose. Samples were collected every 20 second until 2 min, every 1 min until 5 min, and thereafter every 5 min until 30 min. Samples were immediately stocked at −80°C until further analysis. 

### 2.8. Western Blot Analysis of AT1R, IRS-1, IRS-2, PI3-K p85, p-Akt2, GLUT-2, and GCK in Islets

Isolated islets were dissolved in lysis buffer (25 mmol/L HEPES, 50 mmol/L KCl, 6% glycerol, 5 mmol/L EDTA, 5 mmol/L EGTA, 0.5% Triton-X100, 50 *μ*mol/L NaF, 40 mmol/L glycerophosphate, and 25 mmol/L sodium pyrophosphate with proteinase inhibitors). Total protein was measured (BCA protein assay, Pierce, Rockford, IL), and 50 *μ*g protein were fractionated by SDS-PAGE and electrophoretically transferred onto nitrocellulose membranes (Invitrogen). Membranes were incubated in blocking buffer (1TBS, 0.1% Tween 20, and 5% nonfat dry milk) for 1 h at room temperature. The following primary antibodies were used: rabbit anti-AT1R antibody, anti-insulin receptor substrate 1 antibody, anti-insulin receptor substrate 2 antibody, anti-PI3-kinase p85 *α*, anti-phospho-Akt2, anti-glucokinase antibody, anti-glucose transporter- 2 (GLUT-2) antibody, and anti-*β*-actin antibody (Santa Cruz Biotechnology, Santa Cruz, CA). After three washes in TBS/0.1% Tween 20, the membranes were hybridized with a horseradish peroxidase-conjugated anti-rabbit immunoglobulin G prepared in goat (Santa Cruz Biotechnology, Santa Cruz, CA) for 1 h at room temperature. After three washes in TBS/0.1% Tween 20, the bands were visualized by enhanced chemiluminescence (Super Signal West Femto; Pierce, Rockford, IL). The intensities of blots were quantified by scanning densitometry and normalized to the values for actin. 

### 2.9. Statistical Analysis

Data are expressed as means ± SD. Statistical analysis was performed with SPSS XI. Data were grouped according to treatment and analyzed by an independent sample *t*-test or a one-way ANOVA. A value of *P* < 0.05 is considered statistically significant for all comparisons.

## 3. Results

### 3.1. AT1R Expression in Islets of db/db and db/m Mice

**Figure 1:**
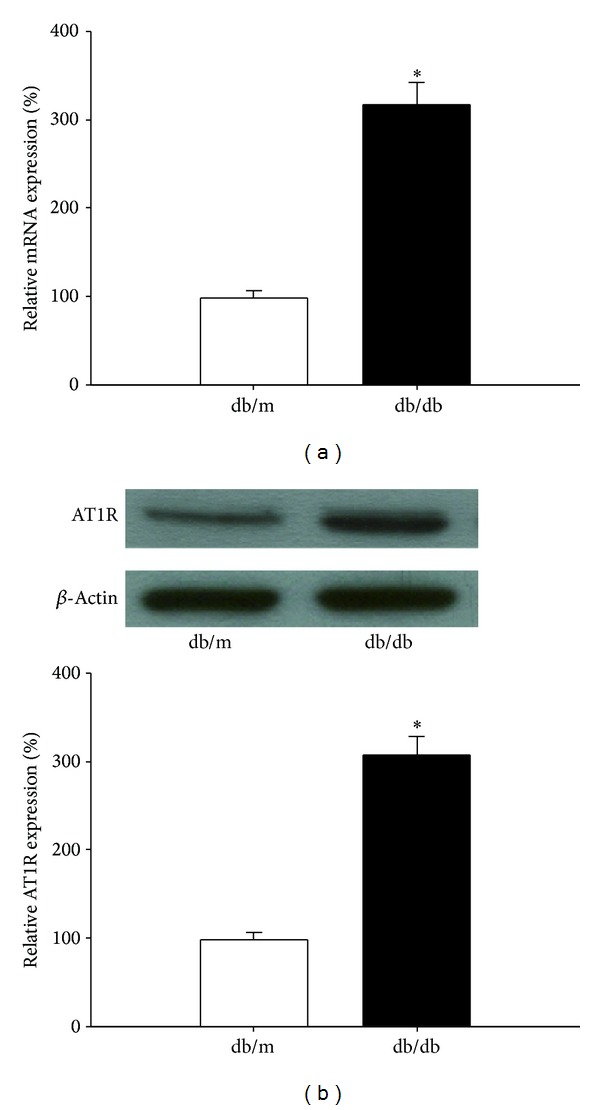
Expression in islet of AT1R in db/m mice and db/db mice. (a) Expression in islets of AT1R mRNA in db/m mice or db/db mice. Gene expression was measured by quantitative RT-PCR. Graphical presentation shows the relative AT1R mRNA abundance after normalization to actin. Data are presented as mean ± SD, *P < 0.05 versus db/m group, *n* = 5. (b) Expression of AT1R protein in islets from db/m mice or db/db mice. Lysates from freshly isolated islets were analyzed for AT1R expression by western blot analysis. Results represent the mean ± SD. Blots from one representative experiments are shown. *P < 0.05 versus db/m group, *n* = 5.

The expression level of AT1R, both in mRNA and in protein, in isolated islets of db/db mice was nearly two times higher than that of db/m mice (*P* < 0.05), indicating that AT1R was overexpressed in diabetic pancreatic islets (Figure 1). 

### 3.2. Reduction of AT1R by Ad-shRNA-AT1R Treatment

**Figure 2:**
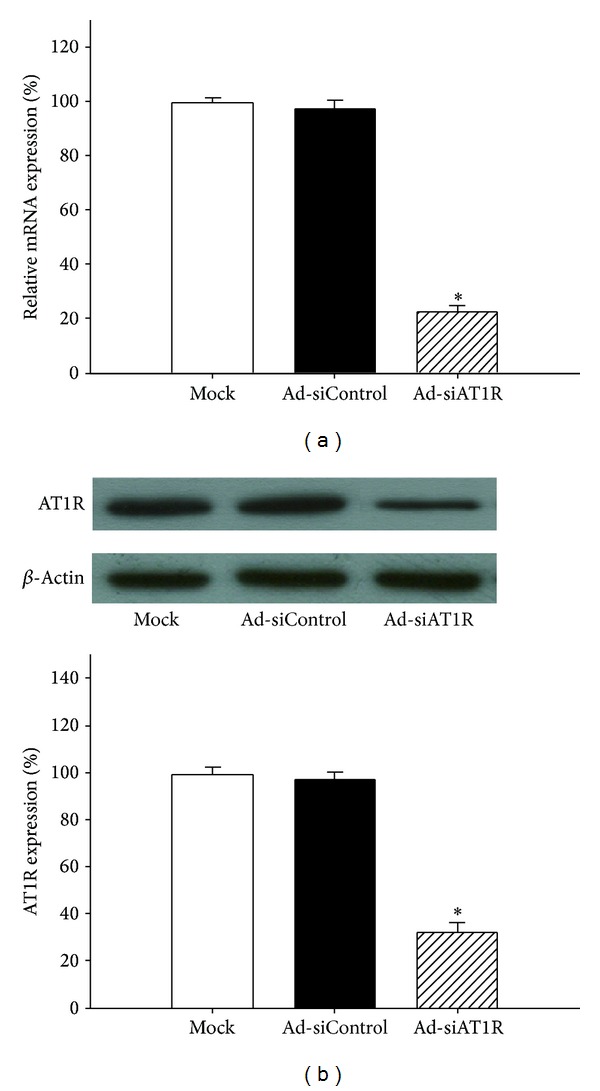
Effects of AT1R silencing on AT1R expression. (a) Islets of db/db mice were transfected with Ad-si AT1R, Ad-siControl, or Mock and cells were cultured for 72 h prior to quantitative RT-PCR. Results are represented as mean ± SD for six independent experiments. *P < 0.05 versus Ad-siControl. (b) Islets of db/db mice were transfected with Ad-si AT1R, Ad-siControl, or Mock and cells were cultured for 72 h prior to quantitative immunoblot evaluation. Results are represented as mean ± SD for six independent experiments. *P < 0.05 versus Ad-siControl.

The islets treated with Ad-siAT1R exhibited a 75% reduction in AT1R mRNA compared with ones treated with Ad-siControl (*P* < 0.05). Moreover, immunoblot analysis demonstrated a 65% decrease in AT1R immunoreactivity in the total extract (*P* < 0.05) (Figure 2). Altogether, these data validated that the RNA interference (RNAi) strategy was effective to suppress the expression of intraislet AT1R.

### 3.3. Improved Insulin Sensitivity in *β*-Cells by Ad-siAT1R Treatment in Islets of db/db Mice

**Figure 3:**
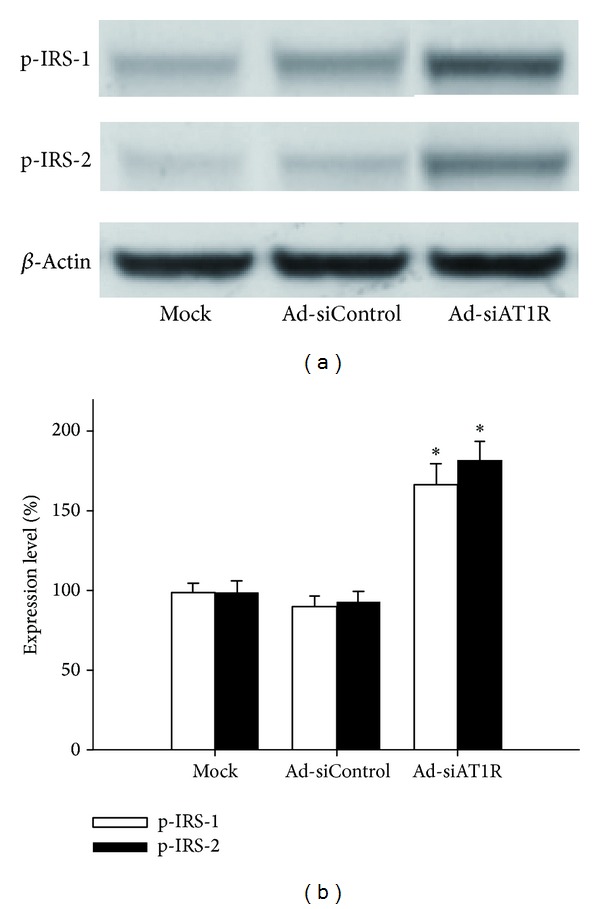
Effects of AT1R silencing on IRS-1 and IRS-2 expression. Islets of db/db mice were transfected with Ad-si AT1R, Ad-siControl, or Mock and cells were cultured for 72 h prior to quantitative immunoblot evaluation. Results are represented as mean ± SD for six independent experiments. *P < 0.05 versus Ad-siControl.

**Figure 4:**
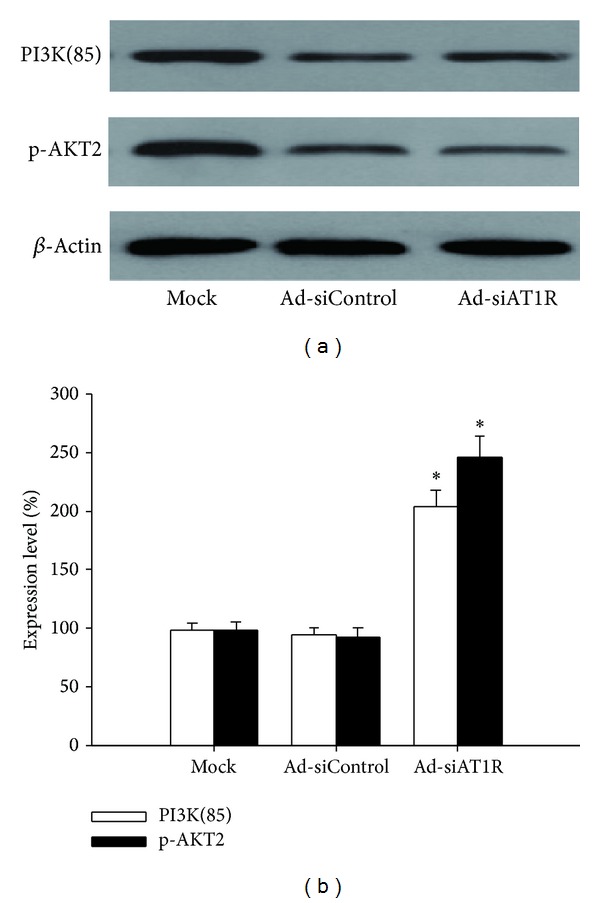
Effects of AT1R silencing on PI3-K(85) and p-Akt2 expression. Islets of db/db mice were transfected with Ad-si AT1R, Ad-siControl, or Mock and cells were cultured for 72 h prior to quantitative immunoblot evaluation. Results are represented as mean ± SD for six independent experiments. *P < 0.05 versus Ad-siControl.

Western blot showed that islets treated with Ad-siAT1R manifested significant increased protein levels of IRS-1 (by 85%), IRS-2 (by 95%), PI3-K(85) (by 112.5%), and p-Akt2 (by 164%) when compared with ones treated with Ad-siControl (*P* < 0.05) (Figures 3 and 4), which indicated that inhibition of AT1R by RNAi improved insulin sensitivity of *β*-cells.

### 3.4. Improved GSIS by Ad-siAT1R Treatment in Islets of db/db Mice

**Figure 5:**
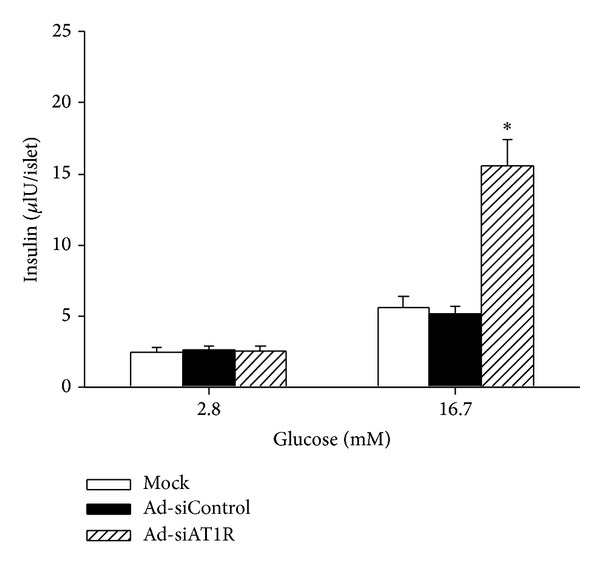
Effect of AT1R silencing on insulin secretion in islets. Islets were treated with Ad-siAT1R, Ad-siControl, or Mock, and 72 h later, insulin secretion was measured at basal and stimulatory glucose. Results are represented as mean ± SD for six independent experiments. *P < 0.05 versus Ad-siControl.

The insulin secretion stimulated by lower (2.8 mM) or higher (16.7 mM) glucose concentration was measured in uninfected cells and cells infected with Ad-siAT1R and Ad-siControl. No significant differences were observed between Ad-siAT1R group and Ad-siControl group at 2.8 mM glucose. Contrarily, there was a significant improvement of insulin secretion response at 16.7 mM glucose (SR 6.1 ± 1.0) in Ad-siAT1R group compared with Mock group (SR 2.3 ± 0.6) or Ad-siControl group (SR 2.0 ± 0.4) (*P* < 0.01) (Figure 5).

**Figure 6:**
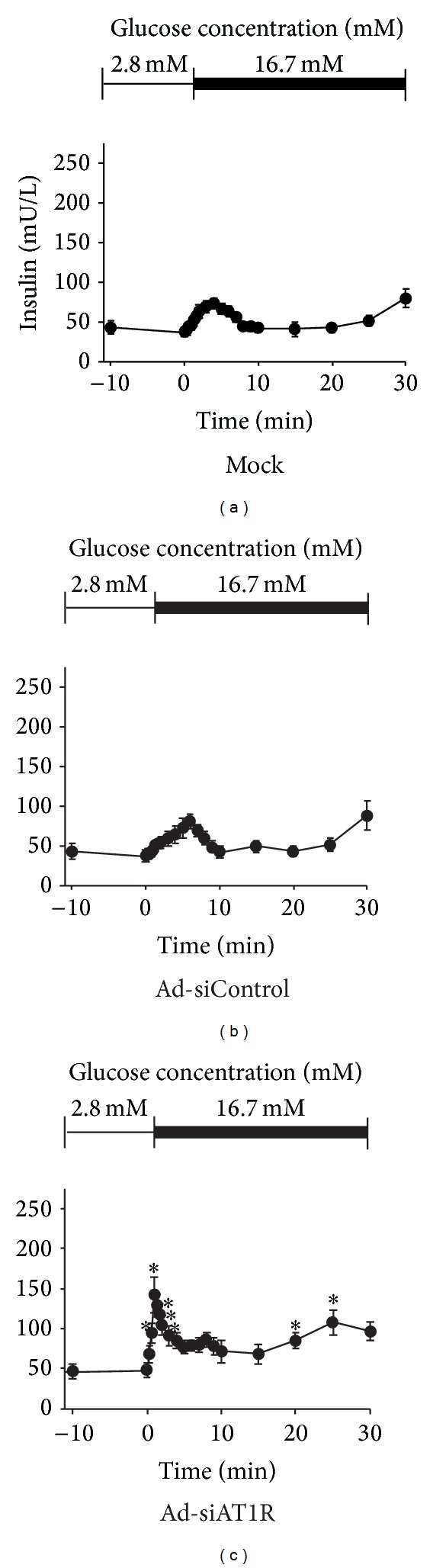
Insulin secretion by islet perifusion in Ad-siAT1R, Ad-siControl and Mock group. Results are represented as mean ± SD for three duplicate experiments. *P < 0.05 versus Ad-siControl.

Perifusion is a golden method to evaluate the first-phase insulin secretion of islet in vitro. Islets of db/db mice manifested only a slight elevation of insulin secretion, while islets treated with Ad-siAT1R showed a pronounced increase in insulin peak at 1 minute after 16.7 mM glucose loaded (*P* < 0.05) (Figure 6).

### 3.5. Reduction of Glucagon Secretion by by Ad-siAT1R Treatment in Islets of db/db Mice

**Figure 7:**
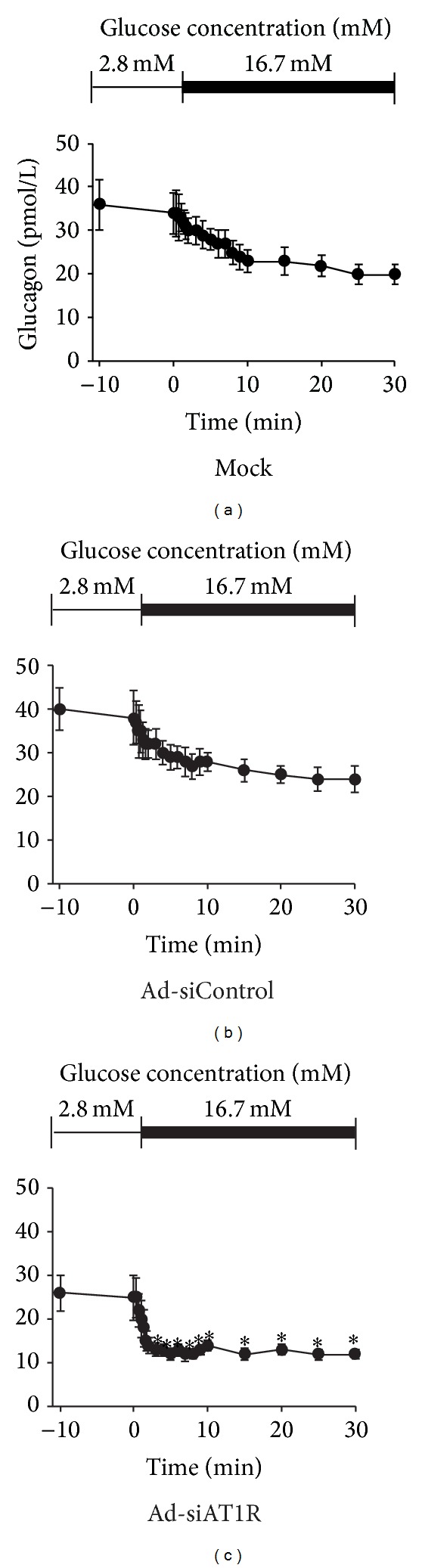
Glucagon secretion by islet perifusion in Ad-siAT1R, Ad-siControl, and Mock group. Results are represented as mean ± SD for three duplicate experiments. *P < 0.05  versus Ad-siControl.

 The Glucagon secretion was assayed in vitro by islet perifusion. Persistently elevated levels of glucagon were observed in Ad-siControl group, while Ad-siAT1R group showed a significant reduction of glucagon secretion since being stimulated by 16.7 mM glucose solution compared with Ad-siControl group (*P* < 0.05) (Figure 7). 

### 3.6. Improved Glucose-Sensing Apparatus in *β*-Cells

**Figure 8:**
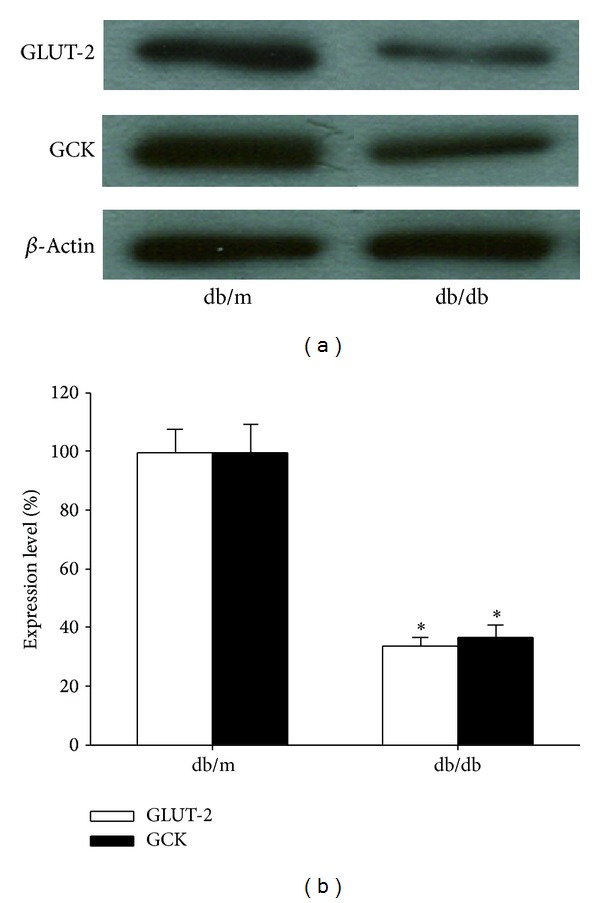
Expression of GLUT-2 and GCK protein in islets from db/m mice or db/db mice. Lysates from freshly isolated islets were analyzed for GLUT-2 and GCK expression by Western blot analysis. Results represent the mean ± SD. Blots from one representative experiments are shown. *P < 0.05 versus db/m group, *n* = 5.

**Figure 9:**
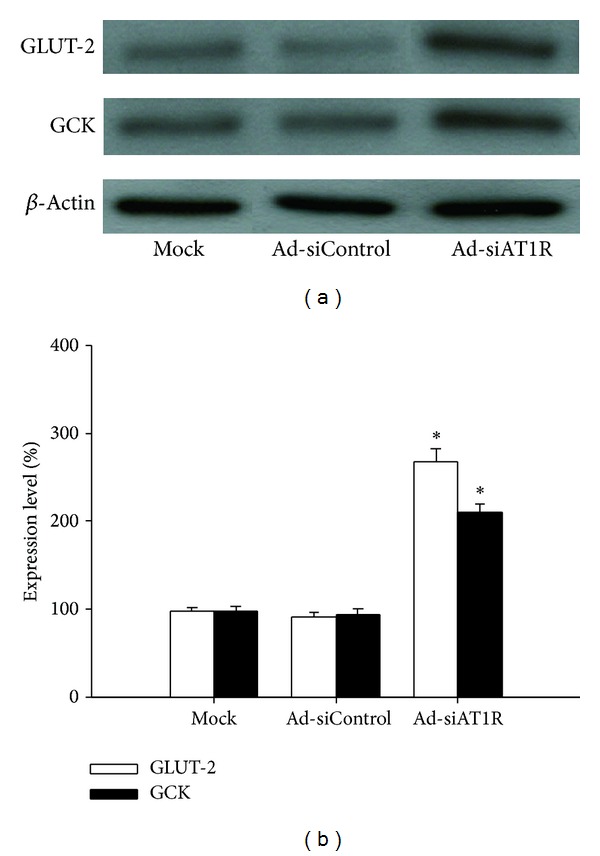
Effects of AT1R silencing on GLUT-2 and GCK expression. Islets of db/db mice were transfected with Ad-si AT1R, Ad-siControl, or Mock and cells were cultured for 72 h prior to immunoblot evaluation of GLUT-2 and GCK. Results are represented as mean ± SD for six independent experiments. *P < 0.05 versus Ad-siControl.

Glucose transporter-2 (GLUT-2) and glucokinase (GCK) have been considered main components of *β*-cell glucose-sensing apparatus. Thus, we further investigated the expression levels of GLUT-2 and GCK in islets. By Western blot, significant decrease in both GLUT-2 (by 65.8%) and GCK (by 62.7%) was found in db/db mice when compared with db/m mice  (*P* < 0.05) (Figure 8). In parallel with the reduction of AT1R expression, the expression of GLUT-2 and GCK increased by 190% and 121%, respectively, in islets treated with Ad-siAT1R, compared with ones treated with Ad-siControl  (*P* < 0.05) (Figure 9). 

## 4. Discussion

 RAS components have long been known to express locally in rodent and human islets. The role of local RAS in islet function has become the focus of recent research, ever since Chappell MC and his colleagues discovered intrinsic angiotensin system in dog exocrine pancreas approximately 20 years ago [19]. The local pancreatic RAS has been shown to be upregulated in diabetic animals, whereas treatment with RAS blockade can improve *β*-cell function and glucose tolerance in variety of studies [20–22]. But the common RAS blockade usually inhibits the RAS systemically rather than locally. So we can not exclude the possibility that these benefits were dependent on the changes of systemic circulating RAS components due to the defects of previous experiment design. In the present study, we indeed inhibited the expression of intraislet AT1R by means of RNAi, a specific and efficient way for gene silencing. We successfully downregulated the expression of AT1R in islet and found not only notable improvement of first-phase insulin secretion but also significant reduction of glucagon secretion in Ad-siAT1R group. What is more, our results showed that improvement of islet function by blocking intraislet AT1R is associated with a detectable increased IRS-1, IRS-2, PI3-kinase p85, and phospho-Akt2 expression levels, as well as increased activities of glucose-sensing apparatus such as GLUT-2 and GCK in pancreatic islet.

The mechanisms of the protective action of RAS blockade on islet function are diverse and complicated. A study found that RAS blockade could improve microvessel density of islets and their function, suggesting that the improvement of blood supply of islets may be a crucial mechanism by which RAS blockade protects islet function [23]. Tikellis et al. [20] showed that chronic (10 weeks) RAS inhibition starting at the age of 10 weeks attenuated disordered islet architecture in Zucker diabetic fatty rats; these beneficial effects were partly attributed to decreased intraislet fibrosis, apoptosis, and oxidative stress. Meanwhile, Kwan and Leung [21] indicated that islet AT1R activation in young diabetic mice could mediate progressive islet-cell failure through UCP-driven oxidative damage. In addition, a recent finding indicated the effects of high glucose levels on islet function might be mediated by local islet RAS, partially AT2 receptors, via the alteration of *β*-cell potassium channels [24]. However, how RAS exerts these effects is less well documented and remains to be clarified.

Several recent studies have indicated that *β*-cells express components of insulin signaling systems including insulin receptors, insulin receptor substrates (IRS-1 and IRS-2), phosphatidylinositol 3-kinase (PI3-K), and protein kinase B [25–27]. IRS-1 and IRS-2 molecules are key mediators in insulin and IGF-1 signaling and their importance in *β*-cell physiology has been proven by several studies. Firstly, insulin binds to receptors on the surfaces of *β*-cells and causes tyrosine phosphorylation of the insulin receptor, IRS-1, and PI3-K. Such activation of PI3-K leads to production of PIP3. Secondly, PIP3 binds with PH domain of Akt and promotes its activation, which stimulates GLUT-2 translocation and glucose uptake into *β*-cells. Finally, increased intracellular glucose leads to increased production of ATP by the catalyze of GCK. The increased ATP/ADP ratio leads to closing of the potassium channel and depolarization of *β*-cells which leads the open of calcium channel and insulin secretion [28]. Therefore, as insulin signal moleculars, IRS-1, PI3-K, and Akt, all play important role in GSIS. Decreased expression of IRSs or derangement in their signal transduction pathway leads to impaired insulin secretion similar to that seen in type 2 diabetes. In this study, we found that inhibition of intraislet AT1R expression resulted in notable improvement of first-phase insulin secretion with significantly increased protein levels of IRS-1, IRS-2, PI3-K p85, and phosphorylated Akt. Such result suggests that IR/IRSs/PI3-K/Akt may act as a potential link between intraislet RAS activity and GSIS. 

As mentioned above, both GLUT-2 and GCK are glucose-sensing apparatus of *β*-cell acting as important roles in GSIS. GSIS is initiated by the uptake of glucose by the translocation of glucose transporter GLUT-2 in pancreatic beta cell. It is suggested that GLUT-2-null mice are hyperglycemic and hypoinsulinemic with a loss of first-phase glucose-stimulated insulin secretion and die within the first 3 weeks of life [29]. Furthermore, GCK is the rate-limiting step in glucose metabolism by *β*-cells, and it therefore has a high control strength over the entire process of glucose utilization, glucose oxidation, and insulin secretion. The discovery that GCK gene mutations account for many of the cases of maturity-onset diabetes of youth (MODY) has pointed out the pivotal role played by this enzyme in glucose homeostasis [30, 31]. Im Walde et al. have found that the GCK mRNA decreased by 50% in pancreas of diabetic mice compared with normal mice [32]. Here we show that GLUT-2 and GCK expression in db/db mice are significantly lower than that in db/m mice, while being restored after AT1R inhibition by Ad-siAT1R. In summary, we can conclude that AT1R inhibition improves GSIS by restoring *β*-cell insulin sensitivity and downstream glucose-sensing apparatus, while the detailed mechanism remains to be further investigated in future work.

A few researches have revealed the mechanisms of AngII-mediated insulin resistance. AngII could exert its influence on insulin sensitivity via the AT1 receptor in at least three ways. Firstly, AngII directly inhibits tyrosine-phosphorylation of IRS-1 and increases serine phosphorylation of IRS-1 and PI3-K p85 regulatory subunit [33]. Secondly, AngII indirectly dephosphorylates IR and IRS-1 by activating TNF-*α* and protein tyrosine phosphatase (PTP)-1B [34]. Furthermore, AngII-induced oxidative stress impairs activity of PI3-K and its downstream signaling, including AKT2-mediated GLUTs translocation and expression levels of other glucose-sensing apparatus [35]. Therefore, in our study, AT1R inhibition induced increased tyrosine phosphorylation of IRS-1 and PI3-K p85 regulatory subunit may contribute to the improvement of *β*-cell insulin sensitivity. And since our previous study showed the expression levels of oxidative stress markers in islet of db/db mice decreased with candesartan treatment, the alleviation of oxidative stress may be also an impact factor of *β*-cell insulin sensitivity and downstream glucose-sensing apparatus expression.

For the first time, we evaluated glucagon dynamic secretion by perifusion and found significantly reduction of glucagon secretion in Ad-siAT1R group. Glucagon is the principal counterregulatory hormone that opposes insulin action leading to coordinate bihormonal control of glucose homeostasis. Increasing evidence has suggested that increased glucagon secretion is implicated in the development of T2DM [36]. A study just published in July showed that enalapril appeared to reduce hyperglucagonemia in high fat diet-induced insulin resistant mice [37]. But the mechanism by which glucagon secretion is inhibited by RAS block is far from clear. Consistent with an important role for insulin in the *β*-cell, insulin receptor and the insulin-signaling molecules are expressed highly in pancreatic *α*-cells and play an important role in modulating *α*-cell function [38–42]. The insulin receptor defects in the insulin-signaling pathway of pancreatic *α*-cell may contribute to the development of diabetic hyperglucagonemia, manifesting as blunted insulin-stimulated Akt phosphorylation and insulin-suppressed glucagon secretion [43]. Therefore, it is conceivable that AT1R block could attenuate hyperglucagonemia by restoring insulin suppression of glucagon secretion driven by insulin, mainly through improved first-phase insulin secretion (indirect) and insulin sensitivity of *α*-cell (direct). Yet the detailed mechanisms remain to be further explored in future research using islet *α*-cell strain.

 In conclusion, our study suggests that intraislet AT1R is a crucial physiological regulator of insulin sensitivity of *β*-cell and thus affects glucose-induced insulin release. We also found that intraislet inhibition of AT1R expression led to downregulation of glucagon secretion from isolated islets of db/db mice. The characteristics of AT1R inhibitors in both insulin and glucagon secretion could make it a potential novel therapeutics for the prevention and treatment of type 2 diabetes.

## Abbreviations

AT1R: Angiotensin II type 1 receptor
GSIS: Glucose-stimulated insulin secretion
IRS: Insulin receptor substrates
GCK: Glucokinase
GLUT-2: Glucose transporter-2
ACEI: ACE inhibitor
ARB: Angiotensin receptor blocker
AGT: Angiotenogen
PI3-K: Phosphatidylinositol 3-kinase
Akt: Protein kinase B
PDK-1: Phosphoinositide-dependent kinase-1.
